# Erasing the Invisible Line to Empower the Pandemic Response

**DOI:** 10.3390/v13020348

**Published:** 2021-02-23

**Authors:** Nicola Decaro, Alessio Lorusso, Ilaria Capua

**Affiliations:** 1 Department of Veterinary Medicine, University of Bari Aldo Moro, 70010 Valenzano, Italy; 2 Istituto Zooprofilattico Sperimentale dell’Abruzzo e del Molise ‘G. Caporale’, 64100 Teramo, Italy; a.lorusso@izs.it; 3 One Health Center of Excellence, University of Florida, Gainesville, FL 32611, USA; icapua@ufl.edu

**Keywords:** SARS-CoV-2, COVID-19, pandemic response, one health, veterinary virologists

## Abstract

A challenging debate has arisen on the role of veterinary expertise in facing the SARS-CoV-2 pandemic. It seems totally unreasonable that in most countries, veterinary diagnostic and tracing forces were not deployed at the start to perform strategic tasks, which could have mitigated the outcome of this dramatic health emergency. Erasing the invisible line between human and veterinary virology will empower the response to future pandemics.

The 2020 pandemic has highlighted many fragilities of the systems in which we operate and rely on as human beings. One of the crucial areas which has most suffered within the health domain is that of activating pandemic reaction systems which include developing and/or applying novel diagnostics, collecting, managing, processing, and reporting for hundreds of thousands of unprogrammed samples with an imposed turn-around time that may well leave many errors behind, as understaffing and under resourcing become a main driving force.

In most countries, veterinary services have extensive experience in diagnosing and managing transboundary animal diseases, most of which are viral. Most viral pathogens which affect humans are the result of a spillover event from other animals [1]. These include HIV, Ebola, Zika but the most fearful due to their pandemic capacity are influenza A viruses (IAVs) and coronaviruses (CoVs). This evidence should lead us to reflect on Rudolf Virchow’s words in 1858: “Between animal and human medicine, there is no dividing line-nor should there be”. Actually, this line is visible to some humans and is reflected in policy, but is invisible to other animals or to the virus itself. Both virus groups in fact recognise animal reservoirs, mainly represented by birds and bats for IAVs and CoVs, respectively, but they are able to easily cross multiple species barriers jumping to humans directly or through intermediate hosts [2,3]. A large part of the scientific knowledge about the molecular mechanisms underlying the cross-species jumping of these viruses and their spillover to humans has been obtained by an outstanding community of veterinary virologists working in multidisciplinary teams.

It seems totally unreasonable that this invisible line has become a powerful dividing entity. The veterinary services and laboratory force could have been from day one an enormous support to the contact tracing (official veterinarians trace animals and their products all the time) and in sample reception, identification, storing, testing, processing, and reporting in ISO 17025-certified environments. In some countries, this external support has been activated and has been crucial, especially during the second wave [4,5,6].

There is one main aspect that needs to be resolved and this is the handling of human subjects for specimen collection and vaccine administration. It seems that similar to nurses, pharmacists, and dentists, also veterinarians may serve this purpose as they are already trained to swab and inject kittens, canary birds and million-dollar horses—and could include this extra activity in their educational curriculum. In addition, veterinarians manage a multitude of vaccination programs using −70 °C and liquid nitrogen-stored vaccines, such as Marek’s disease vaccine which is administered in billions of doses annually.

Last but not least, SARS-CoV-2 has the proven potential of spilling over into naïve animal species and thus of igniting a panzootic [7]. Such an occurrence could carry even more catastrophic consequences and cannot be ruled out. A multitude of domestic and wild animals have been proven to be susceptible to SARS-CoV-2 infection [8]. Cats and mink are infected after close contact with human patients who are positive for COVID-19 [9,10,11], and they are able to transmit SARS-CoV-2 to contact animals and even humans [8,11]. The spillback from mink to humans is of particular concern also due to the emergence of antigenic variants not fully neutralized by antibodies raised against previously circulating strains [11]. The emergence of multiple new variants is already occurring in humans [12] and must be monitored in animals through extensive sequencing, data sharing and open collaboration efforts.

Pandemics occur on a regular basis and we strive to prevent their emergence and to contain them at early stages. Most times we manage to keep these diseases under control but sometimes we fail. Being equipped with more efficient and multidisciplinary response teams cannot exclude that we will fail in containing the pandemics in the future. However, it can give us a better chance in preventing (or significantly slowing down) the worldwide spread of the next pandemic virus.
